# “Understanding dementia together”: The design, delivery and evaluation of a collaborative, inter-professional dementia workshop for healthcare students

**DOI:** 10.1177/14713012241296173

**Published:** 2024-10-30

**Authors:** Trish O’Sullivan, Niamh Moore, Joseph G McVeigh, Suzanne Timmons, Tony Foley

**Affiliations:** Discipline of Physiotherapy, School of Clinical Therapies, 8795 University College Cork, Ireland; Discipline of Medical Imaging and Radiation Therapy, School of Medicine, 8795 University College Cork, Ireland; Discipline of Physiotherapy, School of Clinical Therapies, 8795 University College Cork, Ireland; Centre for Gerontology and Rehabilitation, School of Medicine, 8795 University College Cork, Ireland; Department of General Practice, School of Medicine, 8795 University College Cork, Ireland

**Keywords:** interprofessional education, dementia, collaborative, healthcare students

## Abstract

**Background:**

A collaborative, multi-disciplinary team input is crucial for the optimal management of the older adult with complex care needs such as dementia. Interprofessional learning (IPL) at undergraduate level can lead to improved collaborative knowledge and skills. The aim of this study was to develop, deliver and evaluate an IPL dementia workshop for healthcare students across 11 disciplines. A secondary aim was to determine whether there is a clinical application of learned knowledge in students who completed the workshop and subsequently underwent clinical placement.

**Methods:**

The design of the IPL workshop aligned with Kern’s map for the development of a curriculum in medical education. The Alzheimer’s Disease Knowledge Scale (ADKS) was used to assess students’ knowledge of dementia pre-and-post workshop, as well as opened-ended questions on role recognition and communication.

**Results:**

A total of 102 students completed the workshop questionnaire, with a follow up of 47 students on clinical placement. There was a statistically significant increase in students’ knowledge and confidence levels in communication with a person with dementia. Students reported positively on the workshop format, the collaborative nature of the workshop, as well as the role of the patient advocate. The follow up of students on clinical placement showed a perceived behavioural change in communication modification.

**Conclusion:**

Our study demonstrates the benefits of an IPL initiative across multiple disciplines, with perceived behavioural change on clinical placement.

## Introduction

Dementia is a global healthcare concern and is one of the greatest healthcare challenges of the 21^st^ century. Over 47 million people worldwide are currently living with dementia, with the number predicted to rise to 132 million by 2050 (World Health Organisation, 2017–2025). The economic cost of dementia for governments, communities, families as well as the person with dementia is enormous, estimated at US$818 billion and predicted to rise to US$2.3 trillion globally by 2030 (World Health Organisation, 2017–2025; World Health Organisation, 2018).

Dementia is a clinical syndrome, characterized by a cognitive decline, that leads to impairment in both physical and functional ability. The combination of multi-morbidity and increasing age means that people with dementia are frequent users of health and social care services and have a high risk of hospital admission (Phelan et al., 2012; Shepherd et al., 2019). A collaborative, multi-disciplinary team (MDT) input is crucial for the optimal management of the older adult with complex care needs such as dementia (Grand et al., 2011; Österholm, Nedlund, & Larsson Ranada, 2022). This proactive team approach enables health care professionals to come together and meet the needs of service users, thus ensuring that care is person centred, integrated and optimum (World Health Organisation, 2018).

It is essential that members of the MDT have the necessary skills and competencies to ensure that best patient care is delivered across multiple clinical settings, from acute to community care. Poor patient outcomes have been well documented, including delayed inpatient discharge and a higher risk of falls and delirium (Geriatric Medicine Research Collaborative, 2019; Timmons et al., 2016). Hospital admission for people with dementia is associated with cognitive and functional decline (Andrews, 2013), as well as higher mortality rates. A lack of awareness and understanding about dementia has led to policy directives and recommendations at both national and international level (World Health Organisation, 2017–2025; The Irish National Dementia Strategy, 2014) advocating for training and education of heath care professionals in dementia care.

Evidence suggests that interprofessional learning (IPL) has benefits at undergraduate level (Annear, Goldberg, et al., 2016; Davison et al., 2019). Definitions of IPL in dementia care vary, but for the purposes of this study, IPL occurs when two or more professionals learn about, from and with each other to enable effective collaboration and improve health outcomes (Barr et al., 2016). The benefits of workshop-based IPL have been documented, leading to improved collaborative knowledge and skills (Jackson et al., 2016; Williams & Daley, 2021). Within these workshops, involving people with dementia and/or their carers further increases learning, as well as confidence and attitudes (Williams & Daley, 2021). Allowing students to engage in IPL pre-qualification enables them to understand the value of a collaborative approach in improving patient care, and this insight may cultivate future collaboration (Davison et al., 2019). Quality interprofessional education that enables collaboration, both in the classroom and in practice, is key to efficient workforce development (Hean et al., 2012). One study, which piloted a five-day, protocol-based IPL initiative at undergraduate level among medical, nursing and paramedic students, found improvements in communication and interprofessional relationships (Annear, Eccleston, et al., 2016). Another study, which explored the efficacy of an online interprofessional dementia case study, found that many students aligned the values of interprofessional practice with the provision of high-quality dementia care (Cartwright et al., 2015). However, neither study had representation across a broad spectrum of healthcare professionals, with representation across three and five disciplines respectively. The provision of high-quality dementia care requires input from multiple disciplines in order to meet the chronic and often complex needs associated with this condition. Therefore, the evaluation of a workshop with student representation across a wide range of disciplines would align with this collaborative interprofessional patient care approach.

Despite the growing body of evidence that details the benefits of dementia IPL pre-qualification, no study so far has developed or evaluated a dementia IPL workshop across a broad range of healthcare professionals. Owing to the complex care needs of a person with dementia, many Health Care Professionals (HCP’s) will be involved at different stages of care. General practitioners are frequently the first point of contact, nurses can offer advanced assessment and care co-ordination, including effective communication with carers and family. Physiotherapy, occupational therapy, and radiography are involved in falls, fractures, mobility assessment and diagnostics. Speech and language therapists play a vital role in communication, as well as audiology, given hearing loss in midlife is now a risk factor for dementia (Livingston et al., 2017). Pharmacists are well placed within the community to provide an important role in the pharmacological management of people with dementia, and dentists can enhance a supportive dental care programme to preserve a person with dementia’s oral health. Finally, paramedics play an important role as first responders, for example to people with dementia who have fallen at home.

The aim of this study was to develop, deliver and evaluate an IPL dementia workshop for healthcare students across a broad range of relevant disciplines.

A secondary aim was to determine whether there was a clinical application of learned knowledge in students who completed the workshop and subsequently underwent clinical placement.

## Methods

To achieve the study aims, a dementia IPL workshop was held with students from 11 HCP disciplines, which, to our knowledge, is the largest IPL dementia workshop that has been developed and evaluated to date. Disciplines represented included medicine, nursing, dentistry, physiotherapy, radiography, radiation therapy, audiology, speech and language therapy, pharmacy, occupational therapy, and paramedicine. Students were subsequently followed up on placement to evaluate the extent to which learning from the workshop was applied in clinical practice. This follow up was via survey using the online survey platform Qualtrics XM, where students were asked specific questions on behavioural change on clinical placement.

This study adopted a social constructivist pedagogical approach. Constructivism is a theory of learning whereby individuals construct new knowledge through the interaction between what they already know and new knowledge (Virginia, 2003). This approach asserts that learning is a social process and involves much more than the acquisition of facts (Barak & Green, 2021). Hean et al. (2012), detailed theoretical insights into IPL and described how social constructivism, embedded within the context of adult learning, can scaffold collaborative IPL to negotiate meaning. A social constructivist approach may include case-based, collaborative and contextual learning (Barak & Green, 2020). The authors integrated these three components during the development of the educational intervention.

## Development of the educational intervention

A steering group was formed in July 2022 which included faculty members from 11 healthcare disciplines within the College of Medicine and Health, at University College Cork, Ireland.

**Figure 1.**
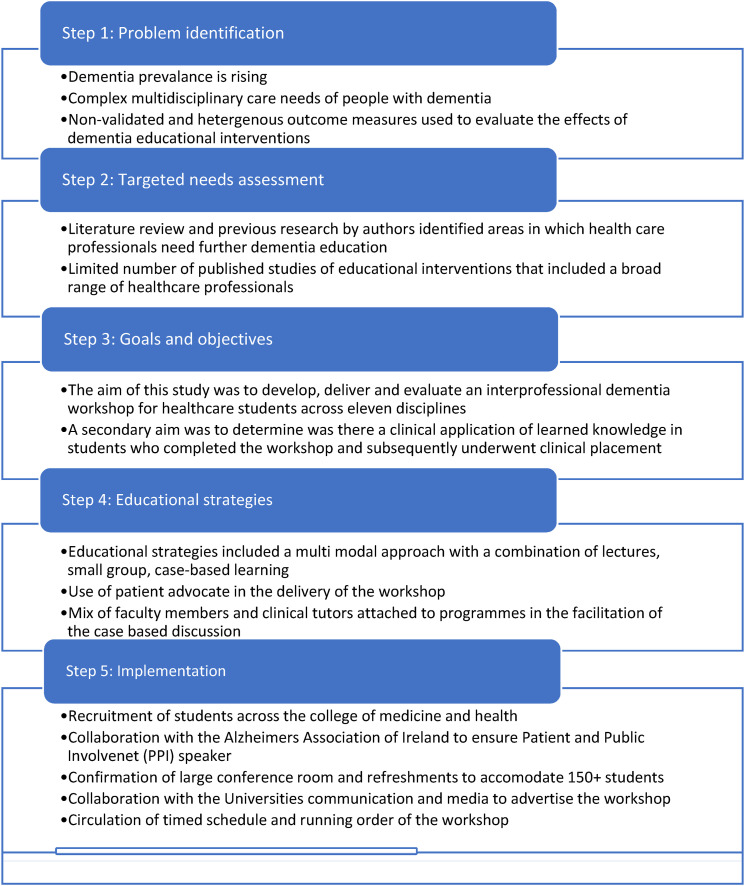
Mapping Kern’s framework to workshop.

Kern’s map for the development of a curriculum in medical education informed the development of the intervention (Thomas et al., 2022). This map is widely used in healthcare education and provides a practical approach to developing, delivering, and evaluating healthcare curricula. Figure 1 illustrates how this model was mapped to the design of the workshop.

**Figure 2.**
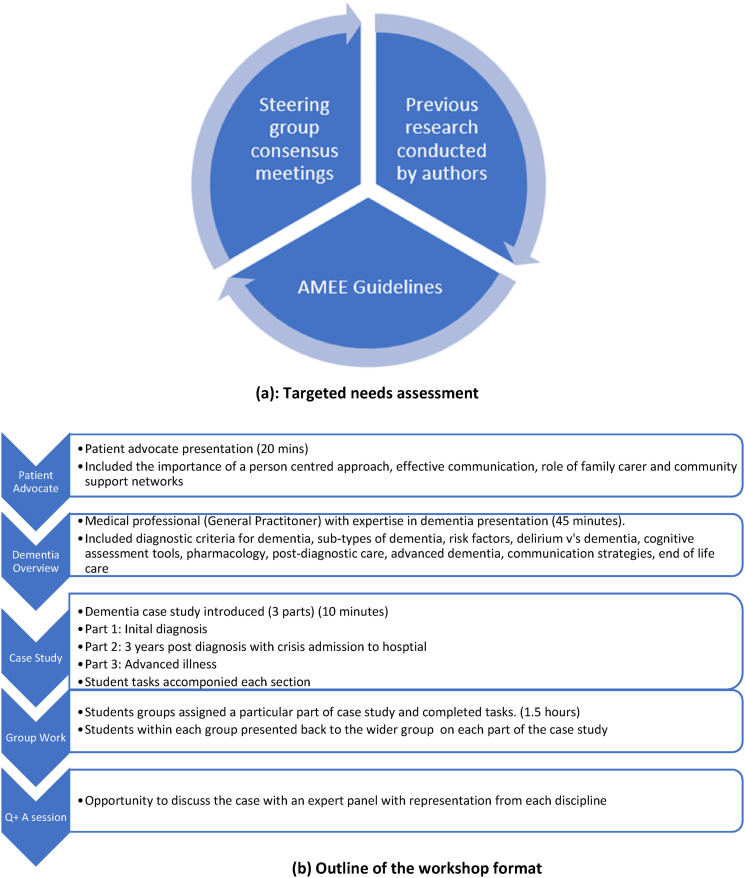
(a) Targeted needs assessment, (b) Outline of the workshop format.

Three steering group meetings were held between July 2022 and October 2022, whereby workshop curriculum content, format and mode of delivery was discussed and agreed upon. This was an iterative process, which included input from all 11 disciplines, to develop a practice-relevant case for all students attending, in line with the adult learning theory (Mukhalalati & Taylor, 2019). The curriculum was also guided by previous research on what constitutes effective dementia education and training, including direct patient/carer involvement and a multimodal approach to teaching and learning (Sullivan et al., 2023). Both these components were incorporated into this workshop. Finally, recommendations from an international association for health professions education (AMEE) guide on theoretical insights into interprofessional education ensured that the workshop adhered to the principles of social constructivism and the adult learning theory, by including a clinically relevant case study, enabling participants to adopt a problem-solving approach, and enabling interaction through group learning (Hean et al., 2012). Figure 2(a) gives an overview of the development of the educational intervention and Figure 2(b) gives an outline of the workshop format.

A dementia case study was created with input from each discipline represented in the steering group (Appendix 1), thus ensuring that learning was contextualised amongst the broad range of disciplines represented. A medical expert in dementia supervised the development of this case study, which followed a patient, from initial diagnosis to a crisis admission to hospital and finally to advanced illness. Thus, multiple clinical settings, from acute to community care and long-term care were represented, reflecting potential scenarios to which students could be exposed on clinical placement, and helping bridge the theory-practice gap. To enhance the fidelity of delivery of the workshop, a facilitators’ guide was developed to accompany the case study, (Appendix 2). This ensured that the workshop was standardised, and could be replicated without reliance on facilitators’ skills, thus reducing unintended variability (Rew et al., 2018). Each discipline contributed to this guide, where it was refined and edited until consensus was reached.

## Ethical approval

The study was approved by the Social Research Ethics Committee, University College Cork (Log, 2020-047A2) and (Log, 2023–131). The participant information leaflet included a data protection notice which outlined where and how long the data was to be stored, how it was to be anonymised and who had access to the data.

## Participants

Healthcare students from all 11 disciplines in the College of Medicine and Health, University College Cork were invited to the workshop. Participants included students from both undergraduate and Master of Science (MSc) (pre-registration) courses. All students were given the opportunity to partake in the voluntary workshop evaluation. In advance of attending the workshop, students were provided with a participant information leaflet informing them about the study. Students used a unique identifier when completing the pre and post workshop questionnaires, thus ensuring anonymity. Table 1 provides a breakdown of disciplines in attendance.

**Table 1.** Breakdown of disciplines.

Discipline	Number of students
Physiotherapy	29
Diagnostic radiography	15
Radiation therapy	8
Occupational therapy	19
Audiology	10
Medicine	8
Nursing	3
Speech and language therapy	28
Pharmacy	18
Dental hygiene	9
Paramedicine	5

## Evaluation of the workshop

The 30-item Alzheimer’s Disease Knowledge Scale (ADKS) questionnaire was used to assess students’ knowledge (Garcia-Ribas et al., 2021). This is a widely used, validated instrument to assess knowledge of Alzheimer’s disease. It measures knowledge across seven domains including risk factors, symptoms, assessment and diagnosis, disease trajectory, life impact, treatment, and caregiving (total possible score is 30). To capture qualitative data on role recognition and communication, a number of questions were included at the end of the ADKS. These questions were piloted by other members of the steering group and refined. Participants were also asked about the positive aspects of the workshop as well as ways improve the workshop.

## Follow up of students on clinical placement

Students in MSc (entry level) Audiology, Radiography, Radiation Therapy and Physiotherapy, who completed the workshop and who were on clinical placement in July and August 2023 were asked to complete a follow up survey, to determine perceived clinical application of learned knowledge, with an aim to reach Kirkpatrick level 3. The Kirkpatrick model is a globally recognised method of evaluating the results of training programs, rating them against four levels of evaluation (Smidt et al., 2009). Level 1 refers to the learner’s response to the training experience. Level 2 evaluates knowledge and/or skills the learner has acquired. Level 3 measures learner behaviour or performance post learning. Three members of the steering group analysed surveys in medical education that aimed to reach Kirkpatrick level three, piloted these questions, and refined them based on feedback, which included re-wording some questions to ensure perceived behavioural change was captured. A copy of this survey can be found in Appendix 3.

## Data analysis

The data from the questionnaire was coded and statistical analyses of paired pre- and post-workshop data for results was carried out using IBM SPSS 28.0 using descriptive and inferential statistics. A paired *t* test was used to determine the change in knowledge pre- and post-workshop. The Wilcoxon signed-rank test was used to determine changes in confidence in communicating with a person with dementia pre-and post-workshop. The significance level was set at 0.05.

Free text comments from the questionnaire were entered into Microsoft word and were independently thematically analysed by two researchers (TO’S and NM) using summative content analysis (Hsieh & Shannon, 2005). Firstly, the authors familiarised themselves with the data. Key features were coded and synthesised into categories. This coding process continued to be refined until consensus was reached between authors. The same method was used in the follow up survey of students who had completed the workshop and who were on clinical placement.

## Results

One hundred and fifty-two students attended the workshop, and 102 students completed the pre-and post-workshop surveys, thereby giving a survey completion rate of 68% of workshop attendees. There was a small but statistically significant increase in students’ knowledge scores post workshop (t (11) = -5.251, *p* ≤ .001), noting a good baseline pre workshop knowledge score, (mean) 23.41, (SD = 2.57); the post workshop knowledge score was (mean) 24.78, (SD = 2.449).

The Wilcoxon signed rank test was performed to compare pre and post workshop confidence levels in communicating with people with dementia. When asked how students felt a person with dementia would understand their explanation of a procedure, the results indicated that students’ median post workshop confidence levels were statistically significantly higher than pre workshop confidence levels Z = −7.584, *p* ≤ .001. The Wilcoxon signed rank test was also performed to compare pre and post workshop confidence in communicating with a person with dementia. There was a statistically significant improvement in confidence post workshop Z = −7.792, *p* ≤ .001.

**Figure 3.**
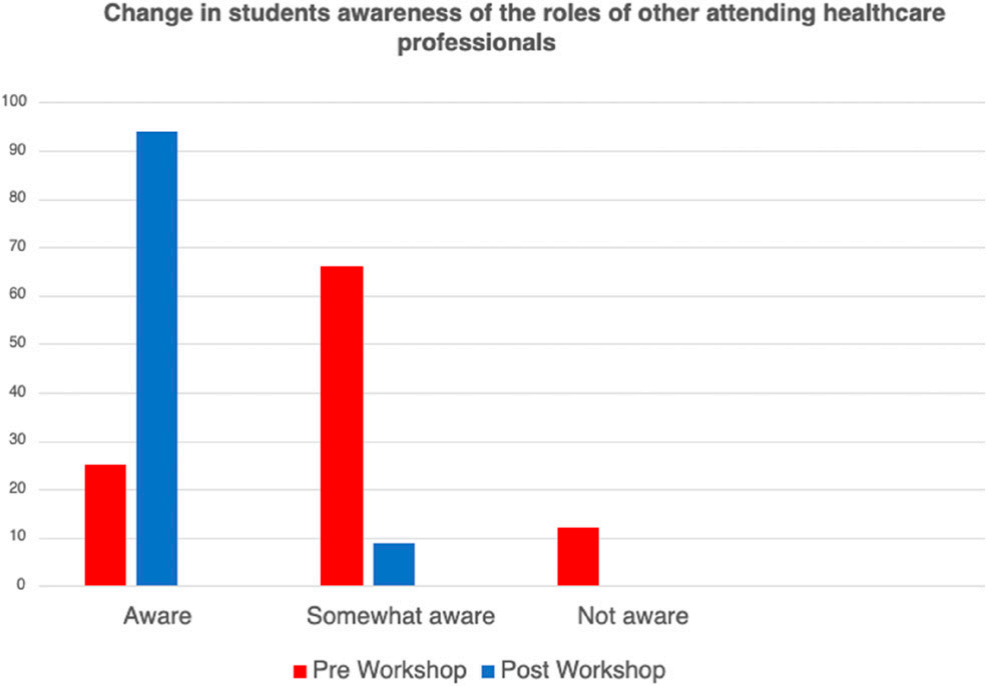
Difference in awareness of other health care professional’s roles pre and post workshop.

Finally, 70% of students felt much more aware of the role of other healthcare professionals after the workshop (Figure 3).

**Figure 4.**
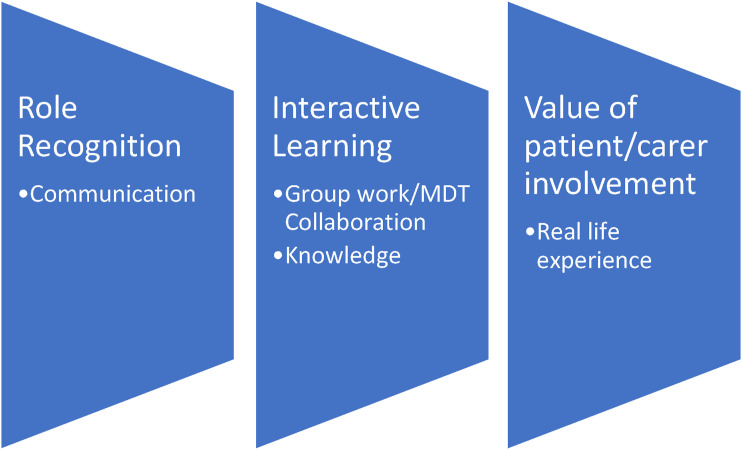
Overview of major and minor themes.

Open-ended questions within the survey explored students’ perceptions about the workshop. Three major themes were identified (Figure 4).

(1) Role recognition
(2) Interactive learning
(3) Value of patient/carer involvement

Within each major theme, one to two minor themes were identified

## Role recognition

The over-arching theme that emerged from the open questions was the value of recognising each discipline’s role in dementia care management. Over half (*n* = 52) cited this as the most positive aspect of the educational workshop. Table two gives an overview of the major and minor themes, with supporting quotes.

### Communication

Students highlighted the value of interprofessional communication and how this platform gave them an opportunity to communicate with each other as well as discussing what communication strategies would be effective in the case scenario.

## Interactive learning

### Group work/MDT collaboration

Students highlighted the value of MDT collaboration and how working together through the case scenario as a team ensured role clarification. The breadth of disciplines represented was valued by students, as were the interesting and differing perspectives offered in discussions around the case study. Some students commented that this was their first experience of MDT collaboration.

### Knowledge

Students outlined how they felt the workshop improved their overall knowledge in dementia. The lecture given by the General Practitioner at the beginning, as well as the case scenario and subsequent group discussion were factors that were perceived to enhance knowledge (Table 2, theme 2.2).

**Table 2.** Major and minor themes with supporting quotes.

Major theme	Minor Theme	Supporting quotes
1) Role recognition	(i) MDT collaboration	“The best part was learning the roles of other members within the MDT”
		“Interesting to see how other disciplines can contribute to the overall plan…integrated, informative and interesting”
	(ii) Communication	“Interprofessional communication to see how others deal with issues in care”
		“Hearing difference perspectives from different professions”
2) Interactive learning	(i) Group work	“Working as a group and hearing individuals’ descriptions of their own role”
		“Discussing with other disciplines about the case”
	(ii) Knowledge	“Putting my knowledge into practice in a hypothetical case”
3) Value of PPI	(i) Real life experience	“Best thing was hearing real life experience, it sticks better”
		“Hearing the perspective of a family member and carer of someone with dementia and their struggle with healthcare”

## Value of patient and public involvement (PPI)

The involvement of a carer of a person with dementia in the workshop, sharing their real-life experience, was viewed by many students as a positive aspect of the workshop, which students reported supported their learning (Table 2, theme 3.1).

## Follow up of students on clinical placement

Forty-seven students from the MSc Physiotherapy, Audiology, Radiation Therapy and Radiography programmes completed the follow up survey while on clinical placement, giving a response rate of 72% (47/65). These students were the only students on clinical placement during July and August 2023, which was nine months after completion of the workshop. Table 3 provides an overview of discipline breakdown and placement clinical area.

**Table 3.** Discipline breakdown and placement site.

Discipline	Number of students	Placement clinical area
Physiotherapy	22	Acute hospital 12
		Private practice 7
		Primary care 1
		Private hospital 1
		Voluntary organisation 1
Radiography	13	Acute hospital 13
Radiation therapy	8	Acute hospital 4
		Private hospital 4
Audiology	4	Primary care 2
		Not specified 2

### Clinical caseload

Most students (91%, 42/46) encountered a person with dementia during their clinical placement, with 78% (32/41) stating that dementia care accounted for between 1%–10% of their caseload, 19% (8/41) stating it was between 11%–30%, and one student stating it was 31%–40%.

### Perceived clinical application of learned knowledge

To capture the extent to which the dementia workshop influenced behaviour while on placement (Kirkpatrick level 3), students were asked three key questions: (1) have you changed the way you communicate with a person with dementia since attending the workshop? (2) have you changed the way you assess and manage a person with dementia? (3) did you learn anything from the workshop that you could confidently apply during your interaction with a person with dementia or their carers?

#### Communication

Most students (79% 31/39) stated that they have changed the way they communicate with a person with dementia since attending the workshop. Three main themes emerged from the open-ended responses: a: Use of simple commands; b. Effective body language; c. Validation.

The use of direct, one stage commands was cited by many students such as, *“I now use calm, short cues”; “Direct, short communication”; “Short, simple effective commands”*.

Concurrent with this change in communication style, was the effective use of body language, with students acknowledging that this is important in establishing a therapeutic relationship with the person with dementia, such as: “…*positive reassuring body language*” and “*I managed to maintain eye contact with the patient”*.

The importance of validation to support meaningful interaction was also highlighted, such as : *“Validating their reality and engaging in meaningful tasks”* and *“Relate to topics in their lives to make them feel more comfortable”*.

#### Assessment and Management

In response to the question on assessment and management, (70%, 26/37) of students stated that they had changed the way they assess and manage a person with dementia since completing the workshop. The was mainly through altered communication, such as: *“Avoid assessments with complex instructions. Keep assessments brief”, and “Simple instructions and valuing the therapeutic relationship over attaining objectives of examinations”*.

#### Application of Learning

In terms of application of learning, here again, the dominant theme was communication. Many students cited modification of their communication strategies as something they have been able to confidently apply during their interaction with a person with dementia during clinical placement, such as: *“Communication skills in order to keep people with dementia at ease and comfortable”; “Speaking slowly, clearly and openly is important”;* and *“How to speak to both the person and their carers and to demonstrate what you want the patient to do”*.

## Discussion

The primary aim of this study was to develop, deliver and evaluate an IPL dementia workshop for healthcare students across eleven disciplines. There was a statistically significant increase in both students’ knowledge of dementia care post workshop, and their confidence in communicating with a person with dementia. The value of role recognition between disciplines, as well as the impact of PPI on learning were predominant themes of the qualitative evaluation. Students also believed that their clinical behaviour had been influenced by the workshop, where students on clinical placement nine months after completion of the workshop felt they had modified their communication and better understood the person with dementia.

Knowledge change from IPL in dementia is commonly assessed, with a range of both validated and non-validated scales used across studies (Dressel et al., 2023; Elvish et al., 2018; Mastel-Smith et al., 2020). In the current study the ADKS was used, which is validated and reliable (Sullivan & Mullan, 2017), but it is not an exhaustive assessment tool (Carpenter et al., 2009). Rather, it contains items that reflect a person’s overall general knowledge on dementia, and it may have a ceiling effect in more specialised groups (Carpenter et al., 2009). An increase in knowledge in dementia is directly correlated with more positive attitudes towards working in dementia care (Hunter & Divine, 2021; Scerri & Scerri, 2013). This was also evident in the follow up survey of students on clinical placement, with students aligning an increase in knowledge with more confidence in dementia care. The improved knowledge of each other’s roles was also evident in both the quantitative and qualitative evaluation of the workshop. This is seen as a positive implication for future collaborative clinical practice.

There was a statistically significant increase in students’ perceived confidence in communicating with a person with dementia following the workshop. Aligned with this, the dominant theme that emerged from the follow up survey of students on clinical placement was the importance of communication modification. Students gave clear examples of how they altered their communication style when engaging with a person with dementia, using simple and unambiguous one stage commands. This illustrates perceived behavioural change, reaching Kirkpatrick Level 3. It is uncommon in healthcare educational literature to evaluate this level, with most studies evaluating to Kirkpatrick Level 1 (reaction) and 2 (knowledge, attitudes, and confidence) (Elvish et al., 2018; Galvin et al., 2010), as found in a systematic review by Surr et al. (2017). A review by Alushi et al. (2015), which evaluated dementia education programs for pre-registration healthcare students, found that no study specifically addressed the need to improve communication skills. Communication deficits such as aphasia, loss of verbal fluency and expression are common in people with dementia (Banovic et al., 2018). Therefore, advanced communication skills training, which encompasses person centred communication strategies, should be an integral component of any dementia educational program.

A dominant theme that emerged from the qualitative evaluation data was the value of PPI. The inclusion of a family carer, who not only gave an insightful presentation of the struggles and challenges of caring for their parent, but also actively facilitated the smaller case-based discussion component of the workshop, was highlighted by many students as a positive aspect of the workshop. The benefits of involving patients within dementia education has been well recognised (Williams & Daley, 2021). Including the direct patient voice and experiences of family caregivers can help in understanding the perspective of the person with dementia, which is a key component in person-centred care (Baille, Sillis, & Thomas, 2106; Surr and Gates., 2017). The ‘patient voice’ is considered a powerful strategy for learning, whereby the learner looks beyond the diagnosis and focuses on the person. The involvement of PPI in dementia education continues to gain momentum, and collaboration between academic institutions and charitable/voluntary organisations are increasing in popularity (Banerjee et al., 2017).

The broad range of disciplines represented in this study is unique, and the value of MDT collaboration was a dominant result in both the qualitative and quantitative evaluation. The inclusion of disciplines such as paramedicine, audiology and dentistry has not been previously evaluated together in the context of dementia IPL before. A systematic review by Jackson et al. (2016), which aimed to identify, describe, and evaluate the impact of dementia IPL, found a maximum of 6 disciplines come together for any one IPL intervention. Thus, the inclusion of 11 disciplines enhanced MDT collaboration across all stages of the case study.

## Implications for future IPL initiatives

The findings of this study provide insight into the optimum modes of design and delivery of an IPL dementia workshop, state of knowledge, confidence, and role recognition across a broad spectrum of healthcare students. The longitudinal follow up of students on clinical placement established perceived behavioural change due to the training, reaching Kirkpatrick Level 3. These findings have the potential to inform future dementia IPL initiatives for a broad spectrum of student healthcare professionals. Each of the 11 discipline representatives on the steering group contributed to the case study. The IPL workshop provided students with clear tasks relating to the case study; students were pre-briefed, and a de-brief session followed the case-based discussion. Pre-allocated student groups were conveyed to the students before the workshop, and students worked predominantly on the case without facilitator input. All these factors have been identified as enablers of student psychological safety, where students feel they can share their thoughts without a fear of rejection, and there is a respect for each other’s capabilities (Lackie et al., 2023).

## Limitations

The generalisability of this study is limited since it was conducted in a single institution. However, the ability to capture such a broad range of disciplines would not have been possible across multiple institutions. Secondly, the follow up of students on clinical placement focused on perceived behavioural change. While self-report can provide some useful information, it can be open to potential bias and effort justification resulting in more positive survey responses. However, owing to the difficulty in quantifying Kirkpatrick level 3, it is usual for studies evaluating medical educational interventions to accept perceived behavioural change as reaching level 3. There was a small, yet statistically significant change in the ADKS, which highlights the ceiling effect of this scale. The dementia knowledge assessment scale (DKAS) has been shown to have less ceiling effect than the ADKS (Annear, Eccleston, et al., 2016) and should be considered in future research. Finally, this workshop was mandatory for some disciplines and not for others, which led to under-representation of some disciplines such as nursing.

## Conclusion

As the number of people diagnosed with dementia each year continues to rise, the provision of high-quality IPL in dementia is a key requirement for the healthcare workforce. Higher educational institutes have a vital role in prioritising collaborative dementia care education and training in undergraduate curricula. Our study demonstrates the benefits of an IPL initiative across multiple disciplines. Continued prioritisation of dementia IPL in HEI’s will ensure that healthcare graduates are equipped to work collaboratively, with appropriate knowledge, confidence and attitudes to ensure the delivery of optimum care of people with dementia.

## Supplemental Material

Supplemental Material - “Understanding dementia together”: The design, delivery and evaluation of a collaborative, inter-professional dementia workshop for healthcare student Supplemental Material for “Understanding dementia together”: The design, delivery and evaluation of a collaborative, inter-professional dementia workshop for healthcare students by Trish O’Sullivan, Niamh Moore, Joseph G McVeigh, Suzanne Timmons, Tony Foley in Dementia: the international journal of social research and practice

Supplemental Material - “Understanding dementia together”: The design, delivery and evaluation of a collaborative, inter-professional dementia workshop for healthcare student Supplemental Material for “Understanding dementia together”: The design, delivery and evaluation of a collaborative, inter-professional dementia workshop for healthcare students by Trish O’Sullivan, Niamh Moore, Joseph G McVeigh, Suzanne Timmons, Tony Foley in Dementia: the international journal of social research and practice

Supplemental Material - “Understanding dementia together”: The design, delivery and evaluation of a collaborative, inter-professional dementia workshop for healthcare student Supplemental Material for “Understanding dementia together”: The design, delivery and evaluation of a collaborative, inter-professional dementia workshop for healthcare students by Trish O’Sullivan, Niamh Moore, Joseph G McVeigh, Suzanne Timmons, Tony Foley in Dementia: the international journal of social research and practice

## Data Availability

In the ethics application, the authors stated that the data would not be shared with anybody outside the research team. Therefore, data will not be uploaded to a repository unless an ethics amendment application is submitted.
