# Supplementary material for: “Understanding dementia together”: The design, delivery and evaluation of a collaborative, inter-professional dementia workshop for healthcare students
Source: Dementia (London). 2024 Oct 30;24(4):720–37. doi: 10.1177/14713012241296173 (PMC11997285; doi:10.1177/14713012241296173)
Supplement: Supplemental Material - “Understanding dementia together”: The design, delivery and evaluation of a collaborative, inter-professional dementia workshop for healthcare student [file sj-pdf-1-dem-10.1177_14713012241296173.pdf]

Dementia IPL Workshop Facilitators Guide

Wednesday October 26th, 2022

Part 1: Introducing Tom

Task 1: Post-diagnostic/Early Management

Coming to terms with a diagnosis of dementia may take a long time both for the person with dementia and their family. The person might experience a range of emotions, including shock, anger, fear, or even relief at having their symptoms explained.

Many people with dementia and their families report not having **access to sufficient information** following a diagnosis. This may be the case however in many cases other factors are involved including person too shocked to take in the information – may need prolonged support from team member, support group, referral for counselling. Too much information given at once – information should be appropriate to the stage of the disease. Information given in a way the person did not understand – recommended that people receive written and verbal information at a level the person understands.

Denial – not conveying the diagnosis and the use of euphemism adds to uncertainty for patients and their families, therefore disclosing a diagnosis is preferable. For some people it may take longer to accept a diagnosis and it may only be achieved after several consultations with the GP. A small number are unable to confront/cope with a diagnosis and in such instances the diagnosis may be withheld. Good team communication is essential at this time.

Practical advice on managing memory deficits might prove helpful at this stage and if the healthcare professional does not feel competent to deliver this information, he/she should have knowledge of local services so that an appropriate referral can be made.

Tom is still independent in all ADL's so this should be encouraged. No issues with mobility or falls has been reported.

Audiology: Age is the strongest predictor of hearing loss in older adults, additionally Tom has a history of prostate cancer. We know that chemotherapy can be ototoxic e.g.Platinum compounds, nitrogen mustard, methotrexate, vincristine, dactinomycin and bleomycin. Therefore referral to Audiology for hearing assessment is appropriate in this case. Additionally, hearing loss is associated with a higher incidence of dementia in older adults.

Early referral here to SLT would be beneficial as they are responsible for assessment, diagnosis & management of acquired communication deficits in addition to eating, drinking and swallowing (EDS) impairments (dysphagia). SLT will also have a role in differential diagnosis, particularly in the diagnosis of Frontotemporal Dementia where communication has been affected (Primary Progressive Aphasia). SLT will have a role in providing education to the family & the patient about to maximise communicative strengths in order to maintain independence in the community. SLT will also have an early role in education to increase the

patient & family's awareness & understanding of dysphagia, how to maintain swallow safety & when swallow review is indicated.

Wife not coping: Linking in with PHN to see if there were any community supports available. Determining the key issues as to why she is not coping. Is it the shock of the diagnosis? Tom is very much independent and it should be encouraged that he carries on wit ADL's as normal for now. Tom's wife may have to assume some household responsibilities like paying bills etc.

Dental perspective:

Oral care needs for a patient recently diagnosed with Dementia.

It is paramount that Tom has a dental examination at this stage to ensure his mouth is in a healthy state and reduce invasive dental treatment at a later stage. Any treatment required such as, fillings periodontal treatment (scaling) sensitivity oral screening-ulcers etc. or denture repairs should be considered as soon as possible.

Aim would be to keep the patients' oral cavity as healthy as possible comfortable and disease freeTom can brush his teeth at this stage as normal but may benefit by a specific routine: aim twice daily and if possible at the same time, an electric toothbrush could be considered.Also be mindful of snacks as food and drinks become a source of comfort and can lead to increase in risk of dental decay. Offer advice on healthy snacks and drinks.

<u>Task 2: What disciplines would be involved at this stage?</u>

-GP, PHN, SLT, Audiology

-Perhaps a joint PT/OT assessment. Advise re importance of exercise at this early stage.

<u>Task 3: Risk Factors</u>

Hearing loss, Ischaemic Heart Disease, Polypharmacy, C_2H_5OH

<u>Task 4: What other differentials should be considered when treating Tom?</u>

Paramedics - consideration should be given to other illnesses which may present simultaneously or mimic AD e.g. infection, stroke, depression and also look out for signs which may be masked by AD such as trauma, paralysis etc.

Part 2: Crisis Admission to Hospital (Three years later)

Task 1 What important information should be included on the referrals for diagnostics?

What information needs to be communicated?
What is relevance and impact of this communication?
What effect can poor communication or omission of information have?

Diagnostic Radiography
Patients are not routinely referred for diagnostic imaging with their notes and very often patients will arrive to for diagnostics such as x-ray/ audiology etc unaccompanied and without any information about their underlying disease, which can cause a great deal of stress for the patient and the staff.

All information about the patient's condition and clinical presentation (eg. not just to outrule #) should be included on the patient's referral letter/(s) as this facilitates the HCP to prepare for a patient with dementia and may involve organising longer appointment time for Tom, less rushing and should involve the patients caring or loved one, who they are familiar and comfortable to be in the company of. The clinician may prepare the room and equipment in advance to accommodate Toms needs and to make it less stressful and more acceptable for Tom. Working with his wife will help the clinician learn the best way to communicate with Tom
The wife is carer and main communication partner, Tom is more relaxed in her company. His should remain in his company where possible, this will more likely result in a diagnostic test and less stressful for Tom.

Task 2 What disciplines would be involved during Tom's admission and time in hospital. Can you discuss each other's role's here?

Medicine: Patient assessment: Establish patient's condition and any underlying conditions or comorbidities that the patient has. Organise for relevant diagnostic tests to be carried out to aid with their diagnosis**.** Organise admission and follow up of patient

Nursing- Admission through ED, Triage of Patient in ED**,** Management of patient and family in ED **Falls Risk** - Visible injuries, Falls risk assessment, cause of fall, home supports, PHN, documentation

Diagnostic Radiography -Comprehensive patient assessment in the Justification and optimisation of the imaging procedure. Assessment of imaging referral to determine most

appropriate modality, and consideration of pt presentation to ensure imaging can be undertaken in accordance with ALARA principles.

Audiology: Hearing assessment -Recognise if the patient has a hearing loss through effective communication. What proactive steps need to be take at this stage? Eg. Discuss with family if there is a confirmed hearing loss and if the patient is a hearing aid wearer. Encourage the family members to bring the hearing aids into the hospital and if necessary organise Audiology appt. with extra time given. Advise on communication strategies, clear masks/face visors and pocket mics during hospital stay.

Audiology: Vestibular Assessment: 80% of fallers have vestibular impairment. Significant age-related depletion of type 1 and 2 peripheral vestibular hair cells (up to 40% in SCC and 25% in otolith organs). Additionally, there is a possibility of vestibulotoxicity based on previous chemotherapy treatment and polypharmacy/previous C2H5OH intake. We would like to rule out a peripheral vestibular impairment as a contributary factor to fall.

Physiotherapy: Falls history- context/characteristics of fall. New baseline. Introduction of aid. Need to lower limb strengthening secondary to de-conditioning. Onward referral to primary care physiotherapy

Occupational Therapy: Falls Planning for discharge home or alternative setting starts immediately with family/carer – goal setting . Assess function, physical and social environment (risk assessment – acute or home setting)Home based assessment of function (if possible). Liaise with family/carer & community service (OT/PHN/GP etc). Assessment – changes – cognitive/sleep/ function/medication/behaviours. Interventions – based on collaborative goals. Home modifications, assistive technology, compensatory strategies/approaches, care giver education, community supports,

SLT: **Discuss re-assessment protocols for communication and swallowing to establish any changes or deterioration. Discuss impact of AD/other medications on communication and swallowing**

- Advancing/progressing presentation of AD
- Medications for AD/other that can impact on swallowing and communication status
- Effects of AD/other medication.
- Modifications to communication and swallowing management plans

Members of MDT to communicate changes in communication and swallowing profile and management (incl. caregiver)

Paramedics;

- Paramedics may be called to Tom's house; if so they should ensure to carry out a thorough patient assessment inclusive of history taking, vital sign recording and any other appropriate tests i.e. point of care glucose, FAST assessment, 12 lead ECG. They should engage and reassure family / carers and determine a plan for Tom

Carry out patient assessment and also report on handover any social conditions or other aspects which help with the continuum of care for Tom. Bring medications or list of same to the hospital and relay what communication / comfort support works best for Tom. Explain to family the plan and reassure where possible.

Dental management:

Oral Care needs for a patient admitted to hospital after a fall with dementia and agitation.

Dental care is not usually required at this stage unless a dental problem is causing problems.

Aim to ensure careers in hospital try to maintain good oral hygiene habits and keep the patients mouth clean to avoid dental problems developing.

Ensure mouth is cleaned either using Electric brush or soft manual, or sponge brush.Mouth rinse Corsodyl if brushing is restricted to limit gingivitis.,

Stress and medication can lead to a Dry mouth products' such as bio-extra rinse can help as well as sipping water regularly.

Diet advice regarding frequency of snacks try healthy snacks and drinks. Use a straw if swallowing is normal if on supplementary food substitutes as these have high levels of sugar.

<u>Task 3 Why do you think Tom is more confused? What could be contributing to his agitated state?</u>

Establish if the patient has an acute delirium (signs to recognise), has Toms conditions deteriorated, is it due to unfamiliar surroundings? Is the patient wearing his hearing devices, can he understand staff communicating with him?

<u>Task 4 What steps could be taken to reduce Tom's anxiety during admission and diagnostics?</u>

- Including Tom in the planning & management of his overall condition
- Ensure all those who encounter any interaction with Tom are aware that he suffers from AD.
- Family member or carer with him initially throughout the process and they can then guide those caring for Tom on communication styles which work with him.
- Prepare the rooms and equipment in advance to make them more AD friendly.
- Organise longer appointment times.
- Family meeting regarding Toms overall understanding and management plan

<u>Task 5: What are the immediate concerns when assessing and treating Tom?</u>

Paramedics: Ensuring that Tom is treated respectfully and appropriately noting that he has AD and also a potential fracture until ruled out. Consider analgesia, monitoring of vitals and

reassessing regularly to ensure nothing is missed. Ruling out other injury and illnesses mentioned earlier.

As relatives are not allowed to travel with Tom, he may be scared and unable to fend for himself so therefore you must advocate more for him.

Part 3: Advanced Illness

Task 1 What are the options for the management of Tom's symptoms?

Discharge team need to look at possibility of LTC v's home. Supports? Ability of wife to be primary carer? Suitable LTC facility with dementia unit? Palliative care involvement due to ongoing hip pain, which is causing Tom distress. An MDT meeting with all disciplines involved in Tom's care would be of benefit here in order to establish a care plan for Tom.

Audiology: Ensure that the correct amplification options are supplied to aid optimum communication. If hearing aids are not appropriate at this time, consider a 'Pocket Talker' which is a personal amplifier. It is easier to use than a hearing aid as the component parts are larger and therefore easier to operate.

Task 2 What ethical issues arise in planning Tom's care?

Ethical issues regarding provision of oral nutrition and aspiration risk – non oral feeding has not been found to improve quality of life in people with advanced dementia but in some cases oral nutrition can place individuals at risk of aspiration. Dietary modification and compensatory strategies (including feeding techniques) may reduce but not eliminate aspiration risk. SLT have a role in providing education to patient, families, and carers in order to determine what is in the patients' best interests. Eating and Drinking with Acknowledged Risk (EDAR) may be appropriate to provide patients with advanced dementia with a means of receiving nutrition, medication and enhance quality of life.

Has Tom appointed an **enduring power of attorney (ETA)?** The attorney may make certain personal care decisions on your behalf - these must be made in your best interests, must be in accordance with what you would have been likely to do and the attorney must consult family members and carers in making these decisions. The attorney is considered to be acting in your best interests if they reasonably believe that what they decide is in your best interests.

Task 3 Who should be involved in Tom's care following discharge home and what might their role be?

Audiology: Advise family members to monitor for any potential deterioration in hearing as hearing aids could be adjusted remotely to increase amplification as needed.

Physiotherapy/Occupational Therapy: If Tom is discharged home, then a joint OT/PT assessment would be warranted. Transfers would need to be reviewed with the home care team. A re-turn (manual aid to help stand Tom) might be useful for toileting.

SLT: Referral to community therapy services on discharge may be indicated for ongoing management of dysphagia (if present). SLT will be able to review swallow to determine

safest consistency in line with IDDSI (International Dysphagia Diet Standardisation Initiative), provide education, particularly regarding aspiration risk and determine course of ongoing management. SLT may also have a role in providing communication strategies & training to families in order to facilitate comprehension & expression in the home environment aiming to ensure that Tom's wishes/choices are followed in relation to everyday decisions as much as possible e.g. likes/dislikes etc.

Nursing: Pressure relief, as well as linking in with the SLT re food intake in light of impaired swallow. Possibility of day care/respite for Tom's wife and family.

Dental measures can become very challenging at this stage as patient may be uncooperative, instruct carer which could be partner or family member or carer on oral hygiene measures, mainly brushing and rinsing. Be mindful wife/family member are under stress, be aware of emotional strain they may be under, be gentle in you approach and don't be critical. Establishing routine is very important, set realistic goals if patient is very agitated don't try and brush at this time wait until they have settled. Wait until patient is settled before trying to brush his teeth, if this cannot be achieve try mouth rinse such as Corsodyl until brushing can be achieved as this can limit the development of gingivitis.

Change to Special care brush example Dr. Barmans' brush which brushes both sides of the teeth at once. Stress and medication or radiation therapy can all contribute to a Dry mouth products' such as bio-extra rinse can help as well as sipping water regularly.

Paramedics may bring him home and therefore ensure that it a place of safety. They should support his family / carers with any immediate queries or questions they may have and outline contact de **Radiation Therapy** Within the care plan potential for pain management RT treatment, including communication and treatment plans in the event of them needing support.
