# Supplementary material for: “Understanding dementia together”: The design, delivery and evaluation of a collaborative, inter-professional dementia workshop for healthcare students
Source: Dementia (London). 2024 Oct 30;24(4):720–37. doi: 10.1177/14713012241296173 (PMC11997285; doi:10.1177/14713012241296173)
Supplement: Supplemental Material - “Understanding dementia together”: The design, delivery and evaluation of a collaborative, inter-professional dementia workshop for healthcare student [file sj-pdf-2-dem-10.1177_14713012241296173.pdf]

Appendix 2: Consent Form

This consent form will appear on the second page of the Qualtrics survey.

By providing my consent below, I agree to take part in this UCC study, which is a follow up study of MSc (physiotherapy, radiography, radiation therapy and audiology) students who completed a dementia inter-professional workshop in October 2022 and who will be on clinical placement in July and August 2023.

I have read the participant information sheet and I clearly understand the potential risks of my participation in this study.

1) I have had the opportunity to ask questions about the study and the questions that I have asked have been answered to my level of satisfaction.
2) I understand that the data I provide will be dealt with in a confidential manner confidentiality will be ensured at all times.
3) I understand that participation in this survey is entirely voluntary and by not participating in this study will not affect my academic grades or grading on clinical placement.

I give my consent to participant in this study: (option to click yes or no on Qualtrics)

Appendix 3: Participant Survey

Title: Understanding Dementia Together: A follow up study of MSc students (physiotherapy. Radiography and Audiology) who completed a dementia inter-professional workshop in October 2023 and who will be on clinical placement in July and August 2023.

Section A: Demographics

What programme are you currently undertaking:

Physiotherapy

Radiography

Radiation Therapy

Audiology

What clinical setting are you currently on placement in?

Acute Hospital Setting

Primary Care

Private Hospital

Voluntary Organisation

Private Practice

Other (Please state)

Section B: Dementia Caseload

Have you encountered a person with dementia during placement since you attended the dementia workshop in October 2022?

Yes

No

If no, please go to Qn_(end of survey)

In what setting have you encountered a person with dementia?

Acute Hospital Setting

Primary Care

Private Hospital

Voluntary Organisation

Private Practice

Other (Please state)

What percentage of your overall daily work includes assessing and managing a person with dementia?

<5%

5-10%

11-20%

21-30%

31-40%

41-50%

51-60%

>60%

Section C: Confidence and Application of Learned Knowledge

Do you feel more confident in managing a person with dementia on clinical placement since attending the workshop?

Yes

No

If yes, in what way?

Do you feel you have more knowledge in the management of a person with dementia since attending the workshop?

Yes

No

If yes, in what way

Have you changed the way you communicate with a person with dementia on clinical placement since attending the workshop?

Yes

No

If yes, in what way?

Have you changed the way you assess and manage a person with dementia on clinical placement?

Yes

No

If yes, in what way?

OR..

Have you used the learning gained from the dementia workshop in clinical practice?

Section C: Inter-professional Collaboration

Have you had an opportunity to work with other disciplines in the management of a person with dementia?

Yes

No

If so, what disciplines?

Do you feel more confident in collaborating with other disciplines on clinical placement since attending the workshop?

Yes

No

If so, in what way?

Thank you for your participation in this survey. If you have any queries about this survey or research, please contact the primary researcher, Trish O' Sullivan, E-Mail: trish.osullivan@ucc.ie
