# Supplementary material for: “Understanding dementia together”: The design, delivery and evaluation of a collaborative, inter-professional dementia workshop for healthcare students
Source: Dementia (London). 2024 Oct 30;24(4):720–37. doi: 10.1177/14713012241296173 (PMC11997285; doi:10.1177/14713012241296173)
Supplement: Supplemental Material - “Understanding dementia together”: The design, delivery and evaluation of a collaborative, inter-professional dementia workshop for healthcare student [file sj-pptx-1-dem-10.1177_14713012241296173.pptx]

## Slide 1
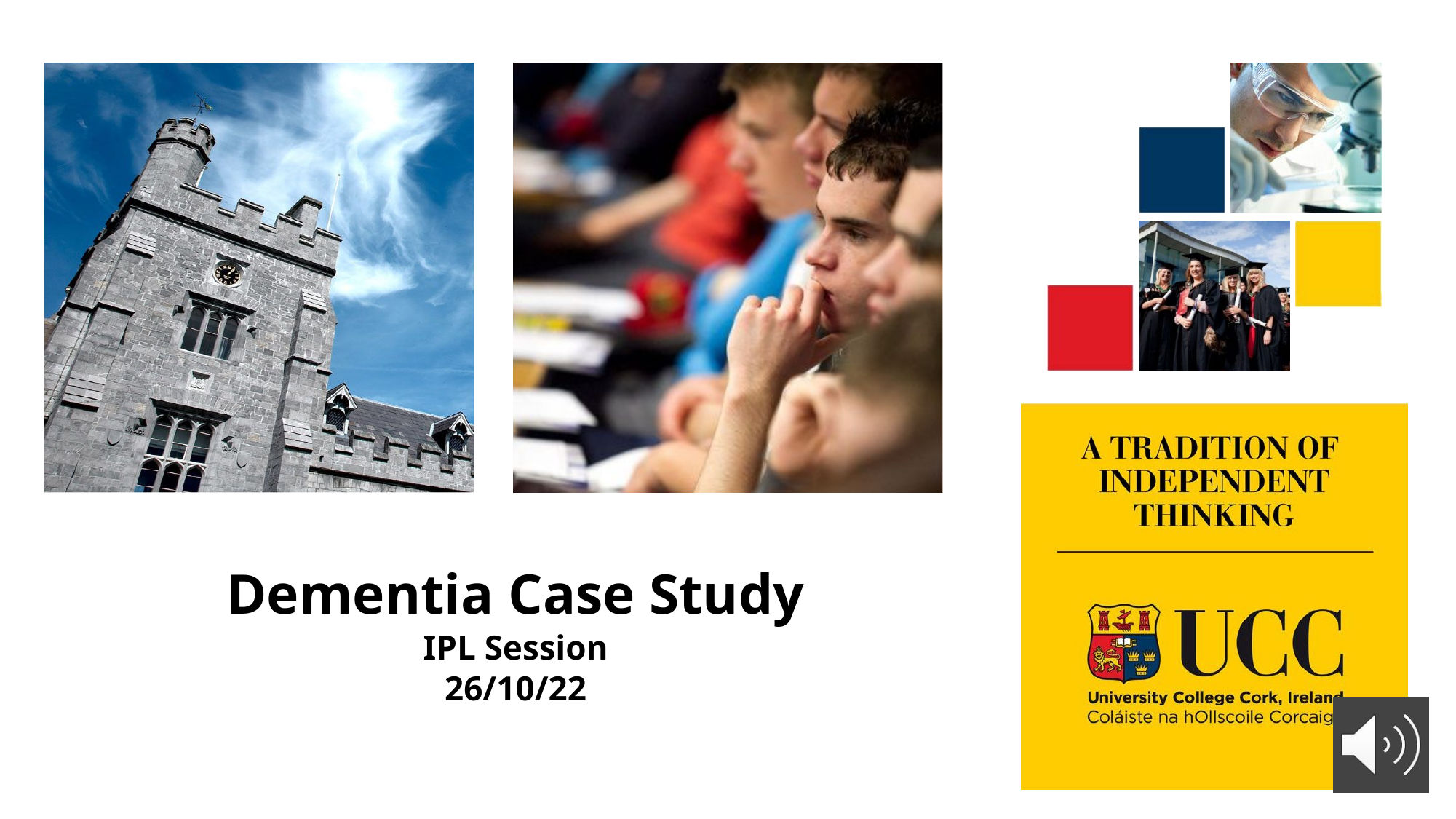

# Dementia Case Study IPL Session 26/10/22

## Slide 2
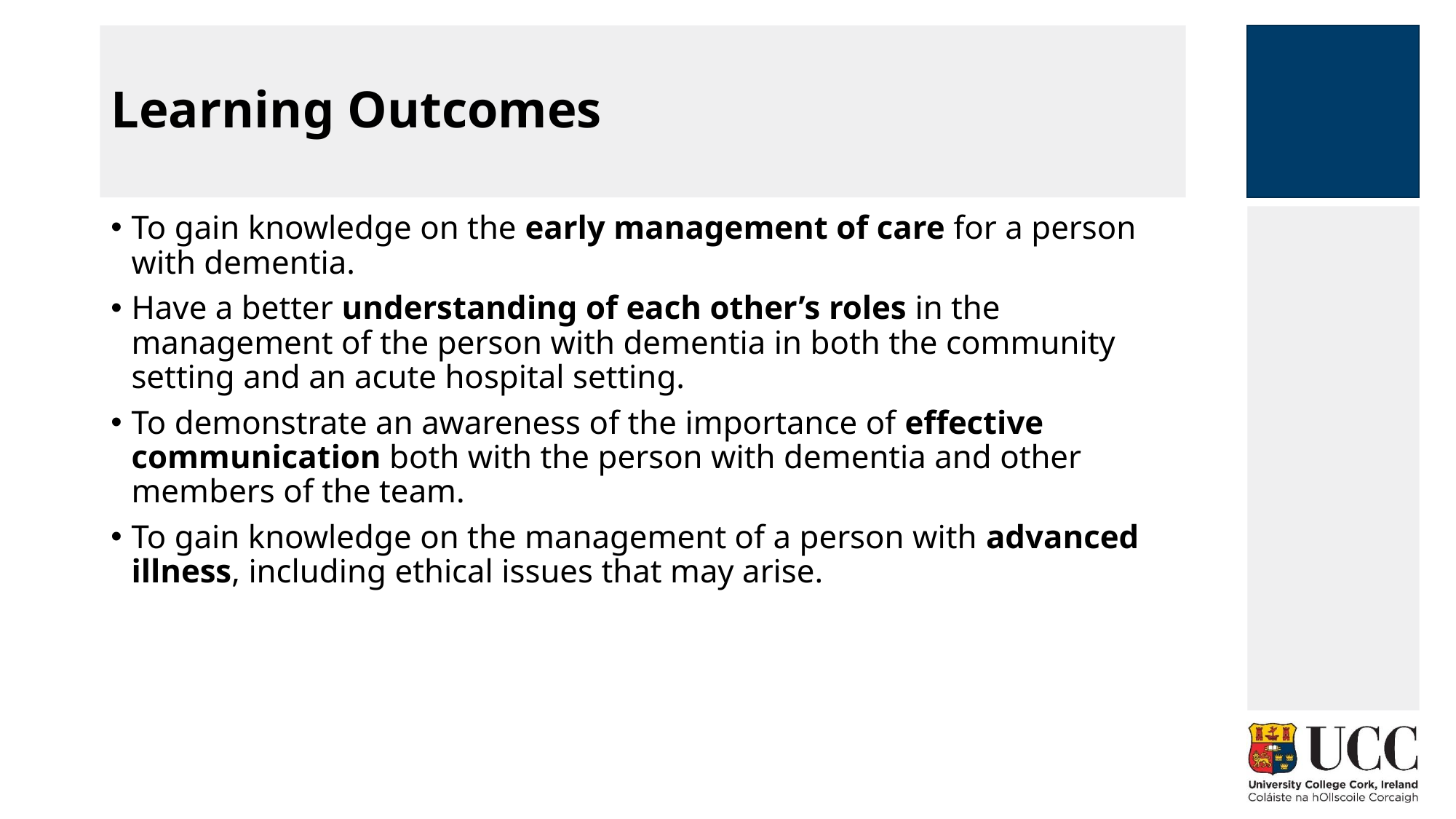

# Learning Outcomes
To gain knowledge on the early management of care for a person with dementia.
Have a better understanding of each other’s roles in the management of the person with dementia in both the community setting and an acute hospital setting.
To demonstrate an awareness of the importance of effective communication both with the person with dementia and other members of the team.
To gain knowledge on the management of a person with advanced illness, including ethical issues that may arise.

## Slide 3
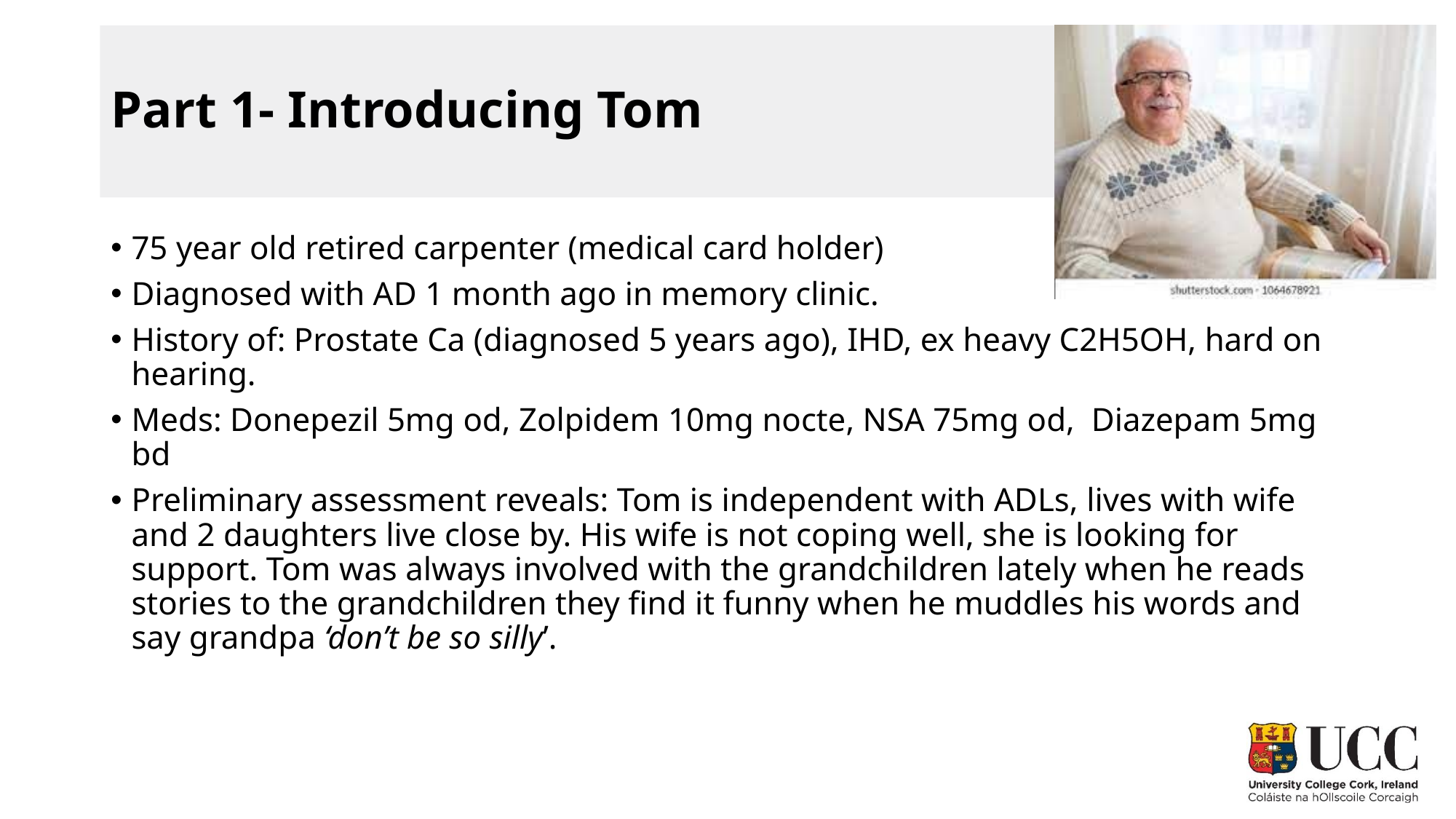

# Part 1- Introducing Tom
75 year old retired carpenter (medical card holder)
Diagnosed with AD 1 month ago in memory clinic.
History of: Prostate Ca (diagnosed 5 years ago), IHD, ex heavy C2H5OH, hard on hearing.
Meds: Donepezil 5mg od, Zolpidem 10mg nocte, NSA 75mg od, Diazepam 5mg bd
Preliminary assessment reveals: Tom is independent with ADLs, lives with wife and 2 daughters live close by. His wife is not coping well, she is looking for support. Tom was always involved with the grandchildren lately when he reads stories to the grandchildren they find it funny when he muddles his words and say grandpa ‘don’t be so silly’.

## Slide 4
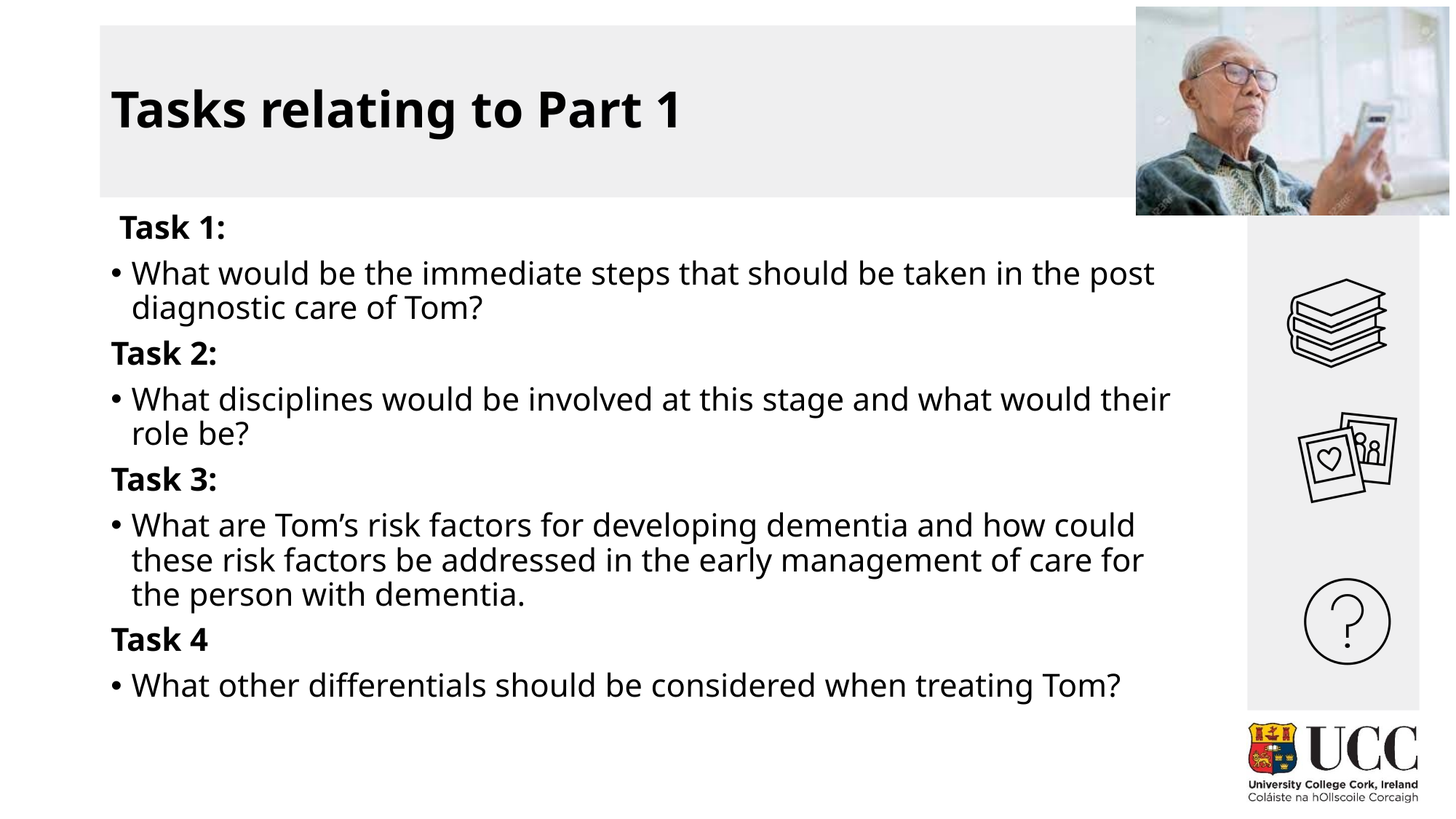

# Tasks relating to Part 1
 Task 1:
What would be the immediate steps that should be taken in the post diagnostic care of Tom?
Task 2:
What disciplines would be involved at this stage and what would their role be?
Task 3:
What are Tom’s risk factors for developing dementia and how could these risk factors be addressed in the early management of care for the person with dementia.
Task 4
What other differentials should be considered when treating Tom?

## Slide 5
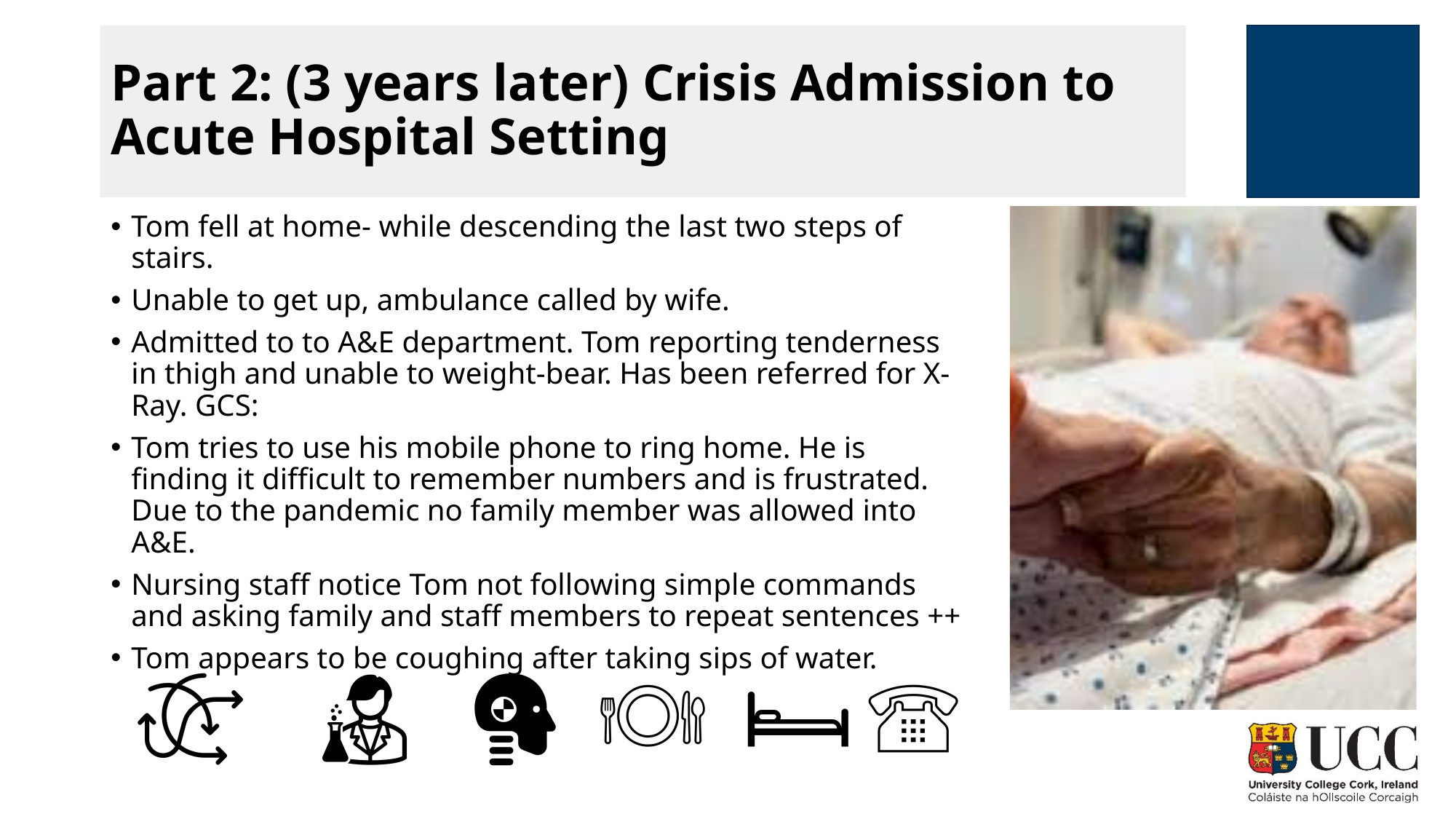

# Part 2: (3 years later) Crisis Admission to Acute Hospital Setting
Tom fell at home- while descending the last two steps of stairs.
Unable to get up, ambulance called by wife.
Admitted to to A&E department. Tom reporting tenderness in thigh and unable to weight-bear. Has been referred for X-Ray. GCS:
Tom tries to use his mobile phone to ring home. He is finding it difficult to remember numbers and is frustrated. Due to the pandemic no family member was allowed into A&E.
Nursing staff notice Tom not following simple commands and asking family and staff members to repeat sentences ++
Tom appears to be coughing after taking sips of water.

## Slide 6
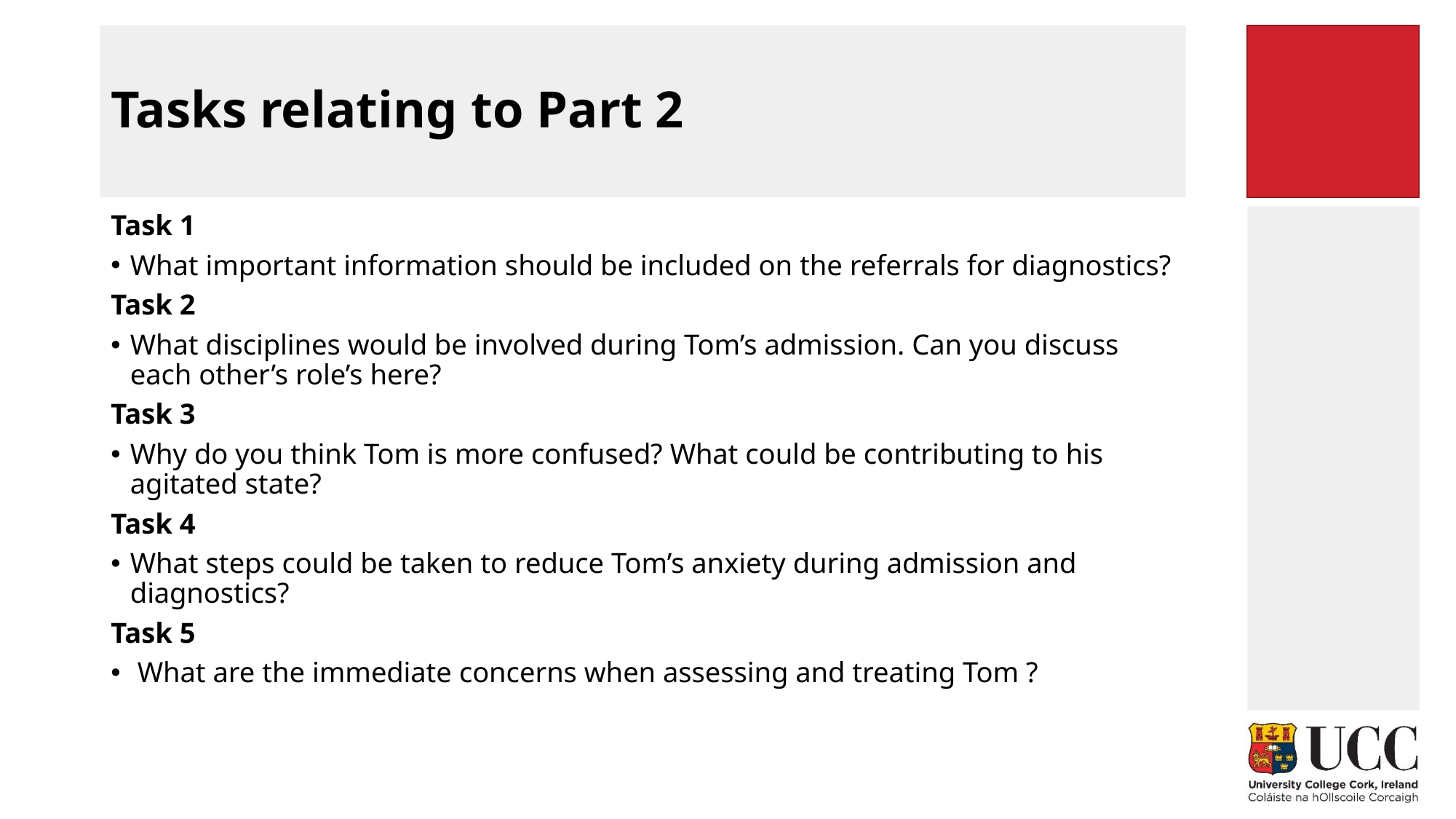

# Tasks relating to Part 2
Task 1
What important information should be included on the referrals for diagnostics?
Task 2
What disciplines would be involved during Tom’s admission. Can you discuss each other’s role’s here?
Task 3
Why do you think Tom is more confused? What could be contributing to his agitated state?
Task 4
What steps could be taken to reduce Tom’s anxiety during admission and diagnostics?
Task 5
 What are the immediate concerns when assessing and treating Tom ?

## Slide 7
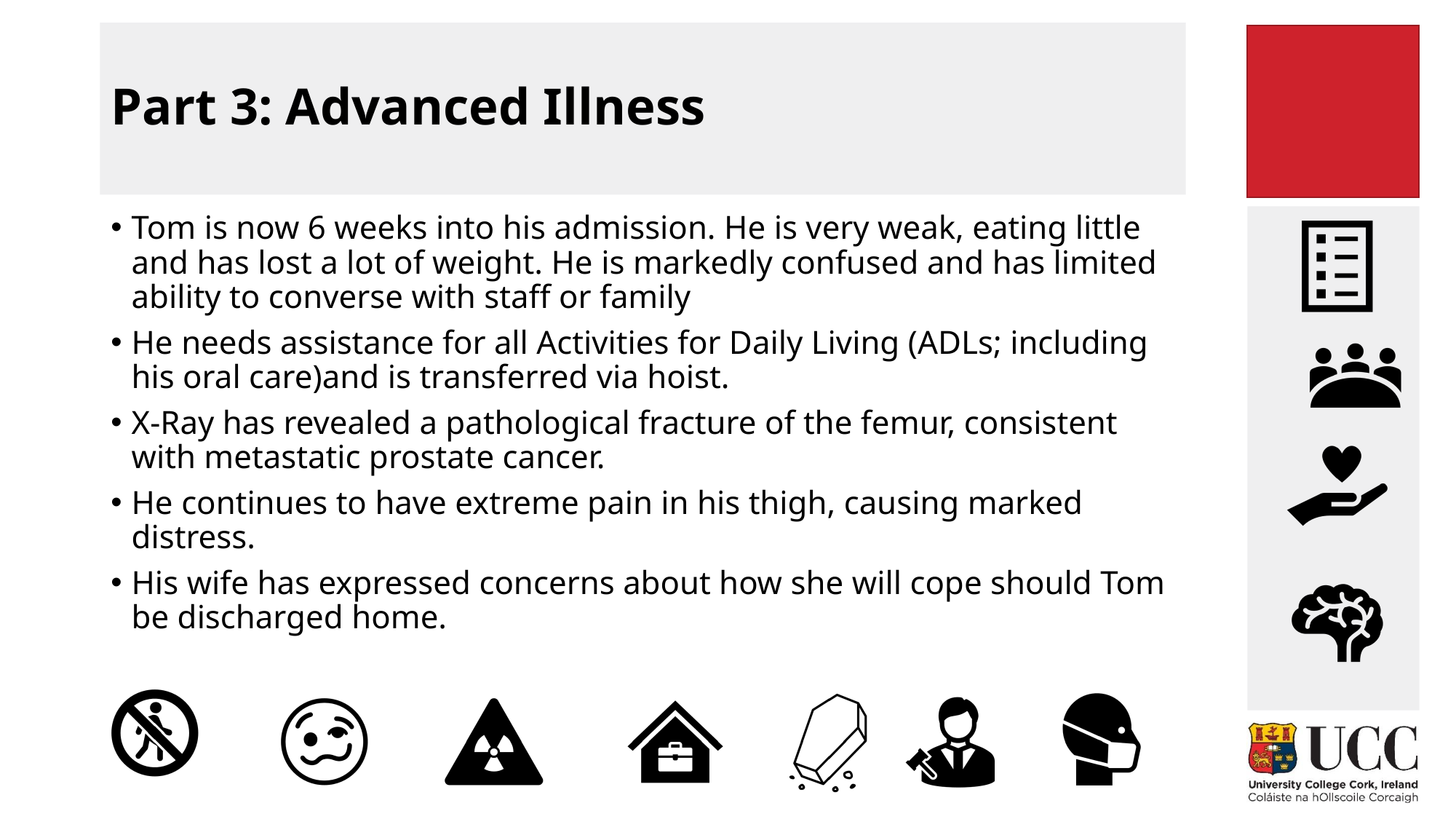

# Part 3: Advanced Illness
Tom is now 6 weeks into his admission. He is very weak, eating little and has lost a lot of weight. He is markedly confused and has limited ability to converse with staff or family
He needs assistance for all Activities for Daily Living (ADLs; including his oral care)and is transferred via hoist.
X-Ray has revealed a pathological fracture of the femur, consistent with metastatic prostate cancer.
He continues to have extreme pain in his thigh, causing marked distress.
His wife has expressed concerns about how she will cope should Tom be discharged home.

## Slide 8
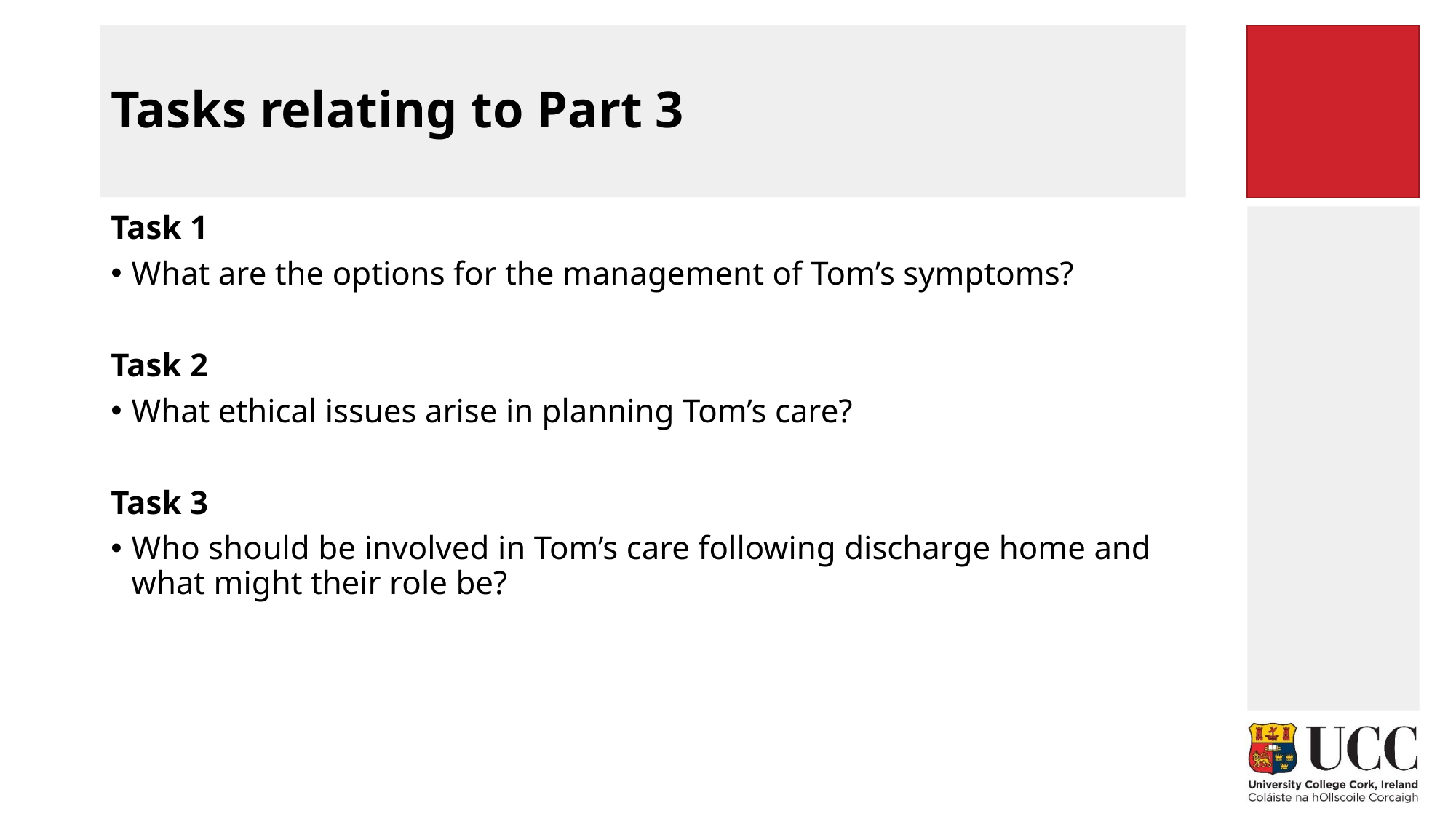

# Tasks relating to Part 3
Task 1
What are the options for the management of Tom’s symptoms?
Task 2
What ethical issues arise in planning Tom’s care?
Task 3
Who should be involved in Tom’s care following discharge home and what might their role be?
